# Genomic characteristics of two breast malignant phyllodes tumors during pregnancy and lactation identified through whole-exome sequencing

**DOI:** 10.1186/s13023-022-02537-w

**Published:** 2022-10-21

**Authors:** Tinge Lei, Mengjia Shen, Xu Deng, Yongqiang Shi, Yan Peng, Hui Wang, Tongbing Chen

**Affiliations:** 1 Department of Pathology, The Third Affiliated Hospital of Soochow University, 213003 Changzhou, Jiangsu P.R. China; 2 Department of Pathology, West China Hospital, Sichuan University, No. 37, Guo Xue Xiang, 610041 Chengdu, Sichuan China

**Keywords:** Malignant phyllodes tumor, Breast, Next-generation sequencing (NGS), Pregnancy and lactation, Molecular profiling

## Abstract

**Background:**

The genomic landscape of breast malignant phyllodes tumors (PTs) is not well defined, especially pregnancy-related malignant PTs. To clarify this topic, whole-exome next-generation sequencing (NGS) was performed on tumor samples and paired normal breast tissues from two pregnancy-related malignant PTs, followed by a functional analysis of the genetic alterations.

**Methods:**

DNA from malignant PT samples and matched normal breast tissues of both patients were subjected to molecular profiling. NGS of the whole-exome was performed in a commercial molecular pathology laboratory. Predictive tools were used to estimate genetic variation in somatic and germline genes.

**Results:**

In total, 29 somatic genomic alterations and 18 germline alterations were found in both patients. In Patient 1, 12 aberrations were identified in the tumor tissue, and 9 alterations were identified in matched normal breast tissue. One pathogenic variant in tumor suppressor genes (*TP53*) was detected in patient 1. In Patient 2, 18 and 10 variants were found in the tumor and matched normal breast tissue, respectively. In Patient 2, pathogenic alterations were identified in two tumor suppressor genes (*PTEN* and *TP53*). *PTEN* and *TP53* may be potential drug targets. The functional predictive tools showed that genes of unknown significance for PTs, including *FCHO1* in Patient 1, and *LRP12* and *PKM* in Patient 2, were pathogenic. Several genes, including *FCHO1*, *LRP12* and *PKM*, were shown for the first time to be altered in malignant PTs. A potentially pathogenic germline variant in *PRF1*, was detected in Patient 1.

**Conclusion:**

Our study first demonstrated somatic and germline gene alterations in two malignant PTs during pregnancy and lactation. These two PTs shared major genetic events, including *TP53* mutation, which commonly occurs in malignant PTs; additionally, we identified two potential genes for targeted therapy, *TP53* and *PTEN*. One germline mutation in *PRF1* was also detected. These results provide clues regarding tumor pathogenesis and precision therapy development.

**Supplementary Information:**

The online version contains supplementary material available at 10.1186/s13023-022-02537-w.

## Introduction

Phyllodes tumors (PTs) are rare breast biphasic neoplasms representing less than 1.0% of all breast neoplasms[1]. Current PT grading includes benign, borderline and malignant PT, based on histological features including stromal cellularity, stromal atypia, mitotic activity, stromal overgrowth and tumor border, as proposed by the 5th edition of the WHO Classification for breast tumors[1]. Malignant phyllodes tumors are usually characterized by marked stromal cellularity, nuclear atypia, stromal overgrowth, more than 10 mitoses per 10 HPF, and infiltrative tumor margins[2]. Current PT grading, according to the above criteria, is based on histological integration of multiple parameters on a semiquantitative basis, which, despite predictive utility across cohorts, cannot accurately ascertain clinical behavior in an individual patient[3].

Malignant PTs account for 10–20% of breast PTs, with a 23–30% local recurrence rate[4]. Up to 25% of cases develop distant metastasis, which may result in patient death[4, 5]. Patients with malignant PTs have a 5-year survival rate of approximately 50% and a 10‐year survival rate of approximately 20%, with a significant association with tumor size and clear surgical margins[2, 6]. Malignant PTs can occur at any age, but the average age at presentation is approximately 40–45 years old[7, 8]. It is rare for young women to have malignant PTs, and even rarer for malignant PTs to develop during pregnancy and lactation[6]. Mastectomy is still the optimal treatment regimen for malignant PTs, combined with radiotherapy and chemotherapy, but the effect of adjuvant therapy remains controversial[6, 9]. The above treatment methods are still adopted for patients with malignant PTs during pregnancy and lactation [6]. However, fatal outcomes of malignant PTs in pregnancy have also been reported with this treatment regimen[10]. Therefore, a deeper understanding of the pathogenesis of such tumors may provide clues for more effective treatments.

With the advent of DNA sequencing technology, the genomic characteristics of breast PTs, including malignant PTs, have been revealed. The genome of malignant PTs had more affected cancer-related genes, such as *NF1*, *RB1*, *TP53* and *PIK3CA*, which were activated as a result of *MED12* mutation or other mechanisms[11, 12]. However, the genomic characteristics of malignant PTs during pregnancy and lactation have not been described, and the factors associated with tumor formation and progression remain unclear. Beyond purely research-driven motivations, the refinement of gene mapping and identification of drug targets may create opportunities for personalized treatment.

Here, we present the gene profiles of two patients with malignant PTs during pregnancy and lactation through DNA sequencing. The indicated aberrations were analyzed for tumors and matched normal breast tissues, which may provide evidence for tumorigenesis and individualized therapy.

## Materials and methods

### Patients

**Fig. 1:**
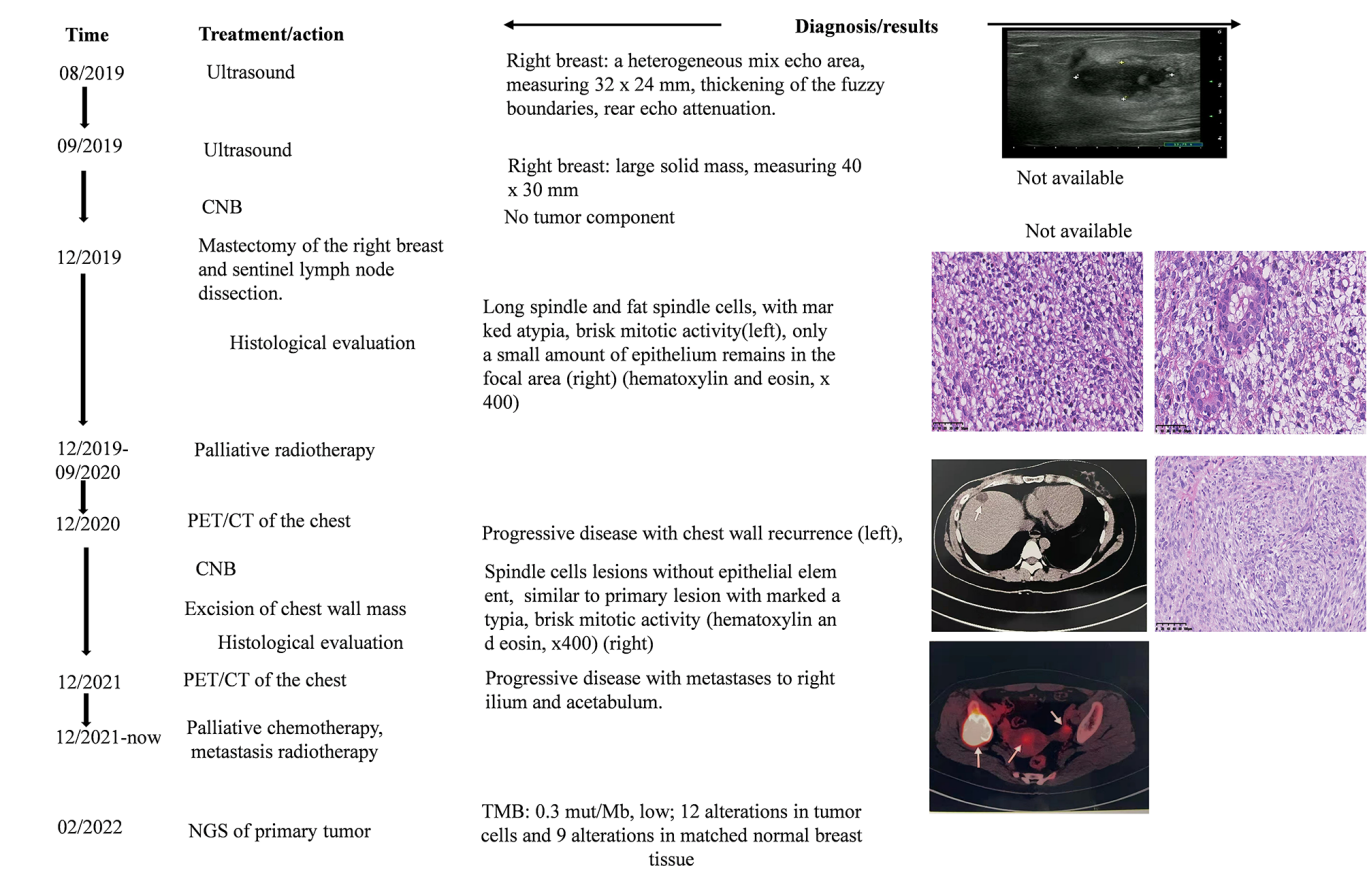
Medical history of a 31-year-old woman with a malignant phyllodes tumor (PT) diagnosed during pregnancy and lactation. The tumor grew rapidly; thus, the patient presented to our institution. She then underwent mastectomy of the right breast and sentinel lymph node dissection in 2019. In 12/2020 and 12/2021, the patient developed chest wall recurrence and distant bone metastasis, respectively. Patient 1 has received chemotherapy and radiotherapy to date. Next-generation sequencing (NGS) of the primary tumor samples and matched normal breast tissue was performed

Patient 1, a 31-year-old female, incidentally discovered a mass on the right mammary gland during early pregnancy. Due to the slow growth of the mass, Patient 1 was not treated during pregnancy, and presented at an institution after delivery. At that institution, breast ultrasound revealed an uneven mixed echo area of the right breast 10 mm from the nipple at the 10 o’clock position with a size of 32*24 mm (Fig. 1). Imaging results suggested a mammary inflammatory lesion during lactation. Therefore, she received traditional Chinese medicine (TCM) treatment. Her symptoms did not improve during the treatment, and she presented at a second institution for an ultrasound examination. Ultrasound (BI-RAS: 4B) showed a solid mass, and a biopsy was recommended. No tumor component was found in the biopsied tissue. The patient continued TCM treatment. During this time, the mass increased and grew more rapidly after weaning; thus, the patient presented at our institution. She had no family history of malignant tumors and was in good general condition. Physical examination revealed a large, firm, regular, palpable mass in the posterior nipple of the right mammary gland from 10 to 2 o’clock. There was no flushing or rupture of the skin and no erosion of the nipple or areola. The patient underwent mastectomy of the right breast and sentinel lymph node dissection. Under macroscopic observation, the tumor was a regular mass 8.5*6.5*5 cm in size. The cross-section of the mass was grayish-white, lobulated and hard. Microscopically, spindle cell tumors do not exhibit heterologous (e.g., liposarcoma, osteosarcoma or chondrosarcoma) components and only a small amount of epithelium remains in the focal area. The diagnosis of malignant PT excluded other spindle cell lesions, especially spindle cell metaplastic carcinoma. In addition, ipsilateral axillary sentinel lymph node examination showed no tumor elements. The patient underwent 25 radiotherapy treatments without chemotherapy after surgery. Three months after the end of radiotherapy, a neoplastic mass was found next to a surgical incision in the right chest wall. Therefore, the patient visited a fourth facility and underwent a positron emission tomography (PET)/computed tomography (CT) examination. PET/CT revealed subcutaneous soft tissue nodules; a core needle biopsy (CNB) of the chest wall tumor was subsequently performed. The pathologic analysis showed spindle cell lesions, which, combined with the medical history, were consistent with the recurrence of malignant PTs. The patient was readmitted to the hospital for chest wall mass resection without adjuvant therapy at the fourth medical institution. Eight months later, a second PET/CT indicated disease progression with metastases to the right ilium and acetabulum. Thereafter, the treatment regimen was changed to systemic chemotherapy (Cariridazul + Etan) plus iliac metastasis radiotherapy, which has continued for eight months, and the patient’s disease has remained stable to date (Fig. 1).

**Fig. 2:**
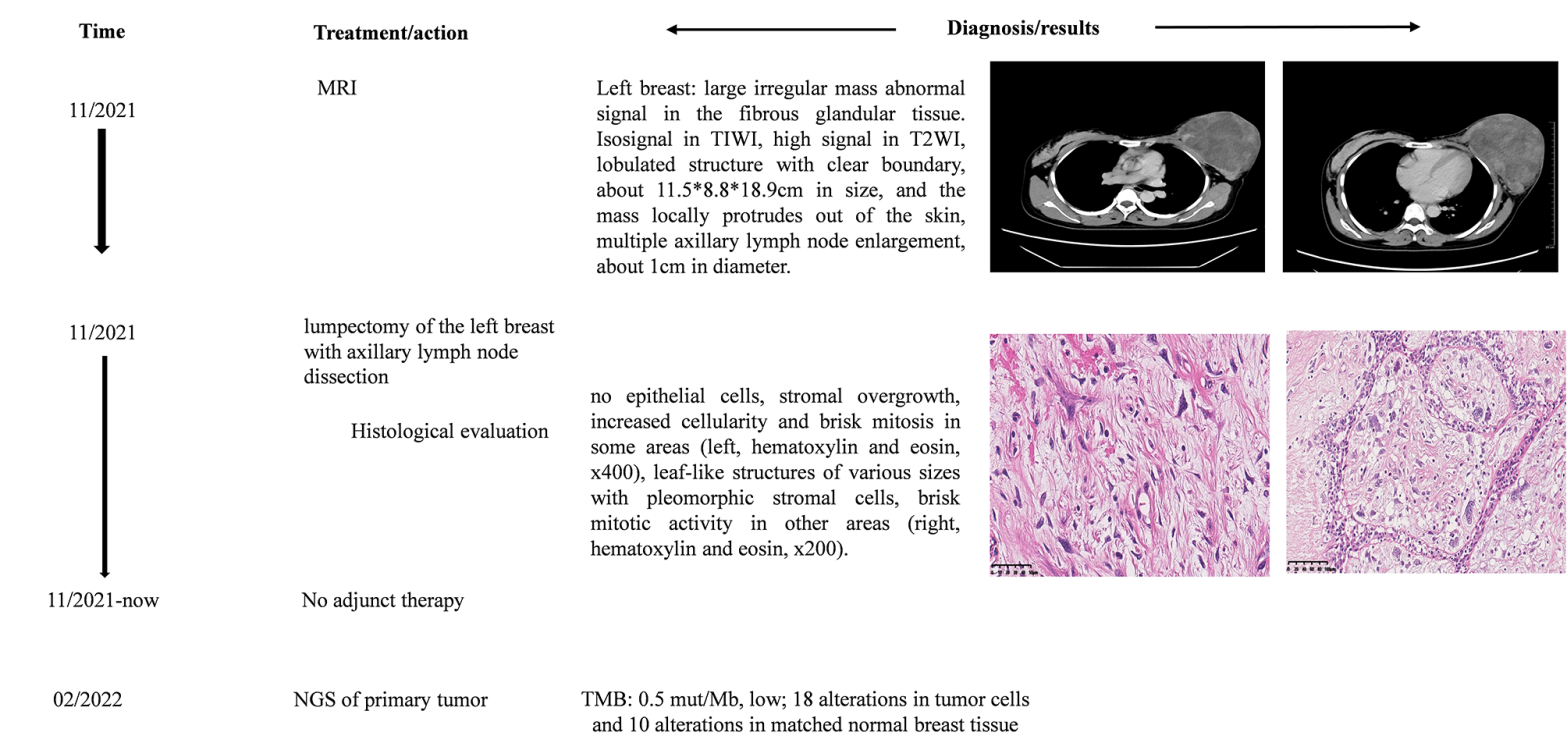
Medical history of a 33-year-old woman with a malignant phyllodes tumor (PT) of the breast during pregnancy and lactation. The young woman presented with a giant mass in her left breast. Lumpectomy of the left breast with axillary lymph node dissection was performed. After surgical excision, no adjuvant therapy was performed. Follow-up was conducted for 5 months, and no signs of disease progression have occurred to date. Next-generation sequencing (NGS) was performed for both the primary tumor samples and matched normal breast tissue

Patient 2, a 33-year-old woman, found a mass in her left breast at the early stage of her second pregnancy. As the pregnancy progressed, the tumor grew and remained untreated. Postpartum, the patient presented at our institution for treatment; at this time, the mass had become the size of a football, tortuous blood vessels could be seen on the surface of the skin, and skin ulcers could be seen in some areas. Magnetic resonance imaging (MRI) (BI-RADS: 4 A) showed a large irregular mass with an abnormal signal, lobulated and clear boundary, approximately 13.5*8.8*18.9 cm in size, almost occupying the entire left breast. After enhancement, the lesions were obviously uneven, with multiple axillary lymph node enlargements, approximately 1 cm in diameter. Subsequently, a lumpectomy of the left breast with axillary lymph node dissection was performed. Gross examination showed that the mass was 18*14*8 cm, and the cut surface was gray, with mucinous changes in some areas and a slightly hard texture. Histological analysis of the surgical specimen showed that the tumor was morphologically heterogeneous, with some areas demonstrating no epithelial cells, increased cellularity and brisk mitosis, and others demonstrating leaf-like structures of various sizes with pleomorphic stromal cells with risk mitotic activity. There were no tumor elements in the axillary lymph nodes. The patient received no adjuvant therapy after surgery, and no recurrence or metastasis was observed over the 5-month follow-up (Fig. 2).

### Ethics statement

According to § 15 of the Nordrhein-Westfalen (Germany) Medical Association professional code of conduct, retrospective studies do not require ethics committee approval. Patients provided written informed consent.

### Genomic profiling

Formalin-fixed, paraffin-embedded (FFPE) specimens of the tumor samples and matched normal breast tissues from both patients were analyzed at a commercial molecular pathology laboratory (Geneseeq Technology Inc, Nan Jing, China). Extracted DNA was subjected to next-generation sequencing (NGS) analysis, namely, whole-exome sequencing (WES). The average coverage depth was over 250X in tumors (98X in normal controls). Library preparations were performed with a KAPA Hyper Prep Kit (KAPA Biosystems). Target enrichment was performed using the xGen Exome Research Panel and Hybridization and Wash Reagents Kit (Integrated DNA Technology) according to the manufacturer’s protocol. Sequencing was performed on an Illumina HiSeq4000 platform using PE150 sequencing chemistry (Illumina). For the targeted panel, customized xGen lockdown probes (Integrated DNA Technologies) targeting 425 cancer-relevant genes were used for hybridization enrichment. The capture reaction was performed with Dynabeads M-270 (Life Technologies) and xGen Lockdown hybridization and wash kit (Integrated DNA Technologies) according to the manufacturers’ protocols. Captured libraries were on-beads PCR amplified with Illumina p5 (5’AAT GAT ACG GCG ACC ACC GA 3’) and p7 primers (5’CAA GCA GAA GAC GGC ATA CGA GAT 3’) in KAPA HiFi HotStart ReadyMix (KAPA Biosystems), followed by purification using Agencourt AMPure XP beads. Libraries were quantified by qPCR using a KAPA Library Quantification kit (KAPA Biosystems). Library fragment size was determined by a Bioanalyzer 2100 (Agilent Technologies). The target-enriched library was then sequenced on HiSeq4000 or HiSeq4000 NGS platforms (Illumina) according to the manufacturer’s instructions. Base substitutions, insertions and deletions, copy number alterations, rearrangements, translocations, microsatellite instability and tumor mutational burden (TMB) were analyzed. The routine result report contained a listing of identified gene alterations.

### Analysis of WES results

All the variants identified (somatic and germline) were clinically classified according to the report of Geneseeq Technology Inc. (InterVar classification). Original image data were transferred by base calling analysis into raw sequence data. Paired-end sequencing data from the exome capture libraries were aligned to the reference human genome (build hg19) with the Burrows‒Wheeler Aligner (bwa-mem)[13]. Single nucleotide variants (SNVs) and short insertions/deletions (indels) were identified by VarScan2 with the minimum variant allele frequency threshold set at 0.01, and a p-value threshold for calling variants set at 0.05 to generate Variant Call Format files. All SNVs/indels were annotated with Annotate Variation (ANNOVAR), and each SNV/indel was manually checked on the Integrative Genomics Viewer. Copy number variation (CNV) analysis was performed using an inhouse developed pipeline. A fold change threshold of 1.6 and 0.6 in DNA copy number was set as the cutoff for amplification and deletion, respectively. Common variants were removed using dbSNP and the 1000 Genome Project. Germline mutations were filtered out by comparison to the patient’s control samples. SIFT[14], PolyPhen[15], and CADD[16] were applied to predict the functional effects of identified genetic alterations. SIFT and PolyPhen (http://genetics.bwh.harvard.edu/pph2/) can be used for the functional analysis of missense variants. PolyPhen and SIFT scores use the same range, 0.0 to 1.0, but with opposite meanings. A variant with a SIFT score of 1.0 is predicted to be benign. A variant with a PolyPhen score of 0.0 is predicted to be benign. SIFT predicts substitutions with scores < 0.05 as deleterious and those ≥ 0.05 as tolerated. A prediction score with PolyPhen ≥ 0.957 is predicted to be probably damaging, between 0.453 and 0.956 is predicted to be possibly damaging, and < 0.453 indicates a benign SNV. CADD is a tool for scoring the deleteriousness of single nucleotide variants as well as insertion/deletion variants in the human genome. It defines phred-like scores (“scaled C-scores”) ranging from 1 to 99, and applies 15 as a cutoff to identify potentially pathogenic variants. In addition, Franklin (https://franklin.genoox.com-Franklin by Genoox) software for the clinical interpretation of variants of unknown significance was also used in our study. Franklin software for the clinical interpretation of variants of unknown significance was also used in our study. Franklin software was the World’s First Open Professional Genomic Community. Franklin software allows genomic professionals to answer almost any genomic question and guides more informed clinical decision-making, facilitating accurate care approaches and enabling more personalized and targeted therapies [17]. Some variants identified through WES have been validated through Sanger sequencing and immunohistochemistry (IHC).

## Results

### NGS of tumor samples and matched normal breast tissue

Alterations in oncogenes or tumor suppressor genes detected in the tumor samples are depicted in Table 1; alterations in genes detected in the tumor samples, which have not yet been clearly identified as oncogenes or tumor suppressor genes, are listed in Table 2.

**Table 1:** Somatic alterations in oncogenes or tumor suppressor genes identified in Patient 1 and Patient 2

Gene	RefSeq NM	Oncogene	Tumor suppressor gene	Type of alteration			Patient	Prediction of functional consequence			franklin	Occurrence of genomic alterations previously reported in phyllodes tumors			
				General classification	Details	Inter Var Class	12	SIFT. pred	PolyPhen. pred	CADD. phred		Specific alteration	Other alterations	Type of tissue	
ATM	NM_000051.3		**+**	Missense variant	p. P2901T	Uncertain significance	1	0 Deleterious	1 D	32 Deleterious	VUS	One case, non- syn. SNV [29]	NR	M PT	
AXIN2	NM_004655.4		**+**	Missense variant	p. T227N	Uncertain significance	1	0.003 Deleterious	0.989 D	25.1 Deleterious	VUS	NR	NR		
BAP1	NM_004656.4		**+**	Missense variant	p. D107E	Uncertain significance	1	0.459 Tolerated	0.015 B	10.98 Tolerated	VUS	NR	NR		
NOTCH1	NM_017617.5	**+**	**+**	Missense variant	p. A1418T	Uncertain significance	1	0.248 Tolerated	0.014 B	15.54 Deleterious	VUS	NR	NR		
TP53	NM_000546.5		**+**	Stop gained	p.E180*	Pathogenic	1	-	-	38 Deleterious	pathogenic	NR, c.747G > C	High frequency [22, 23, 26, 27, 29–34]	Be, Bo, P, R, DM mostly M PT	
				Stop gained	p.C141*	Pathogenic	2	-	-	35 Deleterious		NR, c.747G > C	High frequency [22, 23, 26, 27, 29–34]	Be, Bo P, R, DM mostly M PT	
RB1	NM_000321.2		**+**	Fusion	RB1: exon3~ CORO2B: exon4	-	1	-	-	-	NA	NR, deletion	Several cases [22, 23, 26–34]	Be, Bo, P, R, DM mostly M PT	
FLT1	NM_002019.4	**+**		Missense variant	p. D1140N	Uncertain significance	2	0.277 Tolerated	0.01 B	23.6 Deleterious	VUS	NR	NR		
PTEN	NM_000314.8		**+**	Missense variant	p. D92G	Likely pathogenic	2	0 Deleterious	0.999 P	27 Deleterious	Likely pathogenic	NR	Several cases [23, 26–28]	Be, Bo, mostly M PT	

non-syn. SNV, non-synonymous single-nucleotide variant; NA, not available; P, primary tumor; PT, phyllodes tumor; R, recurrence; DM, distant metastases; Be, benign; Bo, borderline; M, malignan; D: probably damaging; P: possibly damaging; B: benign; VUS: variants of Uncertain significance

The tumor (Patient 1) was microsatellite stable and had a TMB of 0.3 mut/Mb. Twelve genetic variations were identified in the tumor tissue, and nine genetic variants were identified in paired normal breast tissue. Patient 1 had identified somatic alterations in several tumor suppressor genes (*ATM*, *AXIN2*, *BAP1*, *RB1* and *TP53*), and *NOTCH1* with dual roles in activating or suppressing carcinogenesis (Table 1). Among the above alterations, only the *TP53* variant was predicted to be pathogenic. Other gene variants had unknown significance in PTs, and among the genes of unknown significance, only a variant in *FCHO1* was predicted to be pathogenic (Table 2). After the initial diagnosis, the patient received surgery with adjunct radiation therapy. Subsequently, the patient’s treatment regimen was switched to chemotherapy and radiotherapy to the metastases when the disease progressed. She is still receiving this treatment, but has not received NGS-based therapy.

**Table 2:** Variants of unknown significance genes for breast PTs identified in the primary tumor of Patient 1 and Patient 2

Gene	coding(c.) nomenclature	RefSeq NM	franklin	Type of alteration				Patient	Prediction of functional consequence			Occurrence of genomic alterations previously reported in phyllodes tumors		
				General classification	Details	Inter Var Class	12		SIFT. pred	PolyPhen. pred	CADD. phred	Specific alteration	Other alterations	Type of tissue
ARHGEF17	c.5020G > A (p.E1674K)	NM_014786.4	VUS	Missense variant	p. E1674K	VUS	1		0.002 Deleterious	0.549 P	34 Deleterious	NR	NR	
FCHO1	c.2440 A > T (p.K814*)	NM_015122.3	Likely pathogenic	Stop gained	p.K814*	Pathogenic	1		-	-	47 Deleterious	P. R38W c.112 C > T	One case [27]	Be PT
GON4L	c.3970 A > G (p.I1324V)	NM_032292.6	VUS	Missense variant	p. I1324V	VUS	1		0.921Tolerated	0.01 B	0.001Tolerated	NR	NR	
KRT27	c.233G > A (p.G78D)	NM_181537.4	VUS	Missense variant	p. G78D	VUS	1		0.001 Deleterious	0.918 P	26.6 Deleterious	NR	NR	
TGFB1	c.1043 C > A (p.P348Q)	NM_000660.7	VUS	Missense variant	p. P348Q	VUS	1		0 Deleterious	1 D	32 Deleterious	NR	NR	
TTN	c.7721 C > A (p.P2574H)	NM_133378.4	VUS	Missense variant	p. P2574H	VUS	1		0.082 Tolerated	0.74 P	5.255 Tolerated	NR	NR	
CFAP57	c.451 A > T (p.T151S)	NM_152498.3	VUS	Missense variant	p. T151S	VUS	2		1 Tolerated	0.013 B	0.2 Tolerated	NR	NR	
EMILIN2	c.1880del (p.K627Rfs*3)	NM_032048.3	VUS	Frameshift variant	p. K627Rfs*3	VUS	2		-	-	17.26 Deleterious	NR	NR	
F2R	c.280G > A (p.G94R)	NM_001992.5	VUS	Missense variant	p. G94R	VUS	2		0.563 Tolerated	0.01 B	12.59 Tolerated	NR	NR	
IGSF9B	c.2255G > A (p.R752H)	NM_001277285.2	VUS	Missense variant	p. R752H	VUS	2		0.194 Tolerated	0.01 B	24Deleterious	NR	NR	
LILRB2	c.692 C > T (p.P231L)	NM_001080978.4	VUS	Missense variant	p. P231L	VUS	2		0.023 Deleterious	0.325 B	0.623 Tolerated	NR	NR	
LRP12	c.860G > A (p.W287*)	NM_013437.4	VUS	Stop gained	p.W287*	Pathogenic	2		-	-	37 Deleterious	NR	NR	
OR5AN1	c.643 A > G (p.I215V)	NM_001004729.1	VUS	Missense variant	p. I215V	VUS	2		0.304 Tolerated	0.012 B	6.505 Tolerated	NR	NR	
PCDHA13	c.1279G > A (p.A427T)	NM_018904.3	VUS	Missense variant	p. A427T	VUS	2		0.008 Deleterious	0.972 D	26.2 Deleterious	NR	NR	
PKM	c.1516_1525del (p. T506Afs*19)	NM_001206796.3	VUS	Frameshift variant	p. T506Afs*19	Likely pathogenic	2		-	-	34 Deleterious	NR	NR	
RELN	c.6904T > A (p.F2302I)	NM_005045.4	VUS	Missense variant	p. F2302I	VUS	2		0.093 Tolerated	0.996 D	24.6 Deleterious	NR	NR	
SATB1	c.1953T > G (p.I651M)	NM_001131010.4	VUS	Missense variant	P. I651M	VUS	2		0.002 Deleterious	1 D	22.3 Deleterious	NR	NR	
SF3B3	c.2675 C > A (p.A892D)	NM_012426.5	VUS	Missense variant	p. A892D	VUS	2		0.002 Deleterious	0.776 P	32 Deleterious	NR	NR	
SYT16	c.1463 C > T (p.A488V)	NM_001367661.1	VUS	Missense variant	p. A488V	VUS	2		0.217 Tolerated	0.022 B	23.2 Deleterious	NR	NR	
ZSCAN1	c.1094 A > T (p.D365V)	NM_182572.4	VUS	Missense variant	p. D365V	VUS	2		0.083 Tolerated	0.137 B	0.063 Tolerated	NR	NR	
ZXDB	c.2119G > C (p.G707R)	NM_007157.3	VUS	Missense variant	p. G707R	VUS	2		0.045 Deleterious	0.002 B	1.576 Tolerated	NR	NR	

NA, not available; PT, phyllodes tumor; Be, benign; D: probably damaging; P: possibly damaging; B: benign; VUS: variants of Uncertain significance

In Patient 2’s tumor, the TMB was 0.5 mut/Mb and the microsatellite was stable. CNV analysis showed copy number increases on chromosomes 1, 2, and 11 in the tissue samples. Eighteen genetic variations were identified in the tumor tissue, and ten genetic variants were identified in the paired normal breast tissue. Patient 2 had somatic alterations in two tumor suppressor genes (*PTEN* and *TP53*) that were pathogenic (Table 1). Of the unknown significance genes for PTs, variants in *LRP12* and *PKM* were predicted to be pathogenic (Table 2). The patient underwent modified radical mastectomy with no disease progression, and our recommendations have not yet been adopted.

To the best of our knowledge (Tables 1 and 2), sequence variants in 24 genes found in the two patients described here (*ARHGEF17*, *AXIN2*, *BAP1*, *GON4L*, *KRT27*, *NOTCH1*, *TGFB1*, *TTN*, *CFAP57*, *EMILIN2*, *F2R*, *FLT1*, *IGSF9B*, *LILRB2*, *LRP12*, *OR5AN1*, *PCDHA13*, *PKM*, *RELN*, *SATB1*, *SF3B3*, *SYT16*, *ZSCAN1*, and *ZXDB*) have not previously been reported in malignant PTs. The prediction of whether genetic alterations had previously been reported in PTs was based on cited references and PTs listed in the COSMIC database as of 03/2022 (cancer.sanger.ac.uk).

Nine and ten germline variants were detected in the two patients, respectively (Table 3). A possible germinal disease-treating gene, *PRF1*, was detected in Patient 2, but no related studies have been reported to date.

**Table 3:** Germline alterations in genes identified in Patient 1 and Patient 2

Gene	coding(c.) nomenclature	RefSeq NM	franklin	Type of alteration			Patient		Prediction of functional consequence		
				General classification	Details	Inter Var Class			SIFT. pred	PolyPhen. pred	CADD. phred
ABCC2	c.1457 C > T	NM_000392.5	Likely benign	Missense variant	p.T486I	Likely benign	1		0.011 Deleterious	0.063 B	12.73 Tolerated
CYLD	c.98 A > G	NM_001042355.2	VUS	Missense variant	p.K33R	VUS	1		0.321 Tolerated	0.024 B	20.3 Deleterious
EWSR1	c.1493 C > T	NM_005243.4	VUS	Missense variant	p.P498L	VUS	1		0.01 Deleterious	0.001 B	17.89 Deleterious
FOXO3	c.184G > A	NM_001455.4	VUS	Missense variant	p.D62N	VUS	1		0.031 Deleterious	0.229 B	19.65 Deleterious
GRM8	c.1012 A > G	NM_000845.3	VUS	Missense variant	p.I338V	VUS	1		0.916 Tolerated	0.013 B	6.229 Tolerated
LZTR1	c.1766T > C	NM_006767.4	VUS	Missense variant	p.L589P	VUS	1		0.152 Tolerated	0.999 D	25.5 Deleterious
MPL	c.173 C > T	NM_005373.3	Likely benign	Missense variant	p.A58V	VUS	1		0.045 Deleterious	0.533 P	17.63 Deleterious
			Likely benign		p.A58V	VUS		2	0.045 Deleterious	0.533 P	17.63 Deleterious
RCC1	c.1123T > C	NM_001269.5	VUS	Missense variant	p.Y375H	VUS	1		0.57 Tolerated	0.021 B	18.18 Deleterious
TET2	c.4229 C > T	NM_001127208.2	VUS	Missense variant	p.P1410L	VUS	1		0.001 Deleterious	1 D	33 Deleterious
ATM	c.125 A > G	NM_000051.3	Likely benign	Missense variant	p.H42R	VUS		2	0.353 Tolerated	0.057 B	8.953 Tolerated
ERBB3	c.1981G > A	NM_001982.3	VUS	Missense variant	p.G661S	VUS		2	0.323 Tolerated	0 B	7.111 Tolerated
FANCF	c.1009_1014del	NM_022725.4	VUS	inframe_deletion	p.G337_D338del	VUS		2	-	-	22.4 Deleterious
FGFR1	c.103G > A	NM_023110.3	VUS	missense_variant	p.G35R	VUS		2	0.674 Tolerated	0.022 B	21.3 Tolerated
GSTP1	c.439G > T	NM_000852.4	VUS	missense_variant	p.D147Y	Likely benign		2	0.002 Deleterious	0.428 B	23.3 Deleterious
MTRR	c.362G > A	NM_001364440.2	VUS	missense_variant	p.R121Q	VUS		2	0.752 Tolerated	0.003 B	9.307 Tolerated
NOTCH1	c.6788G > A	NM_017617.5	Likely benign	missense_variant	p.R2263Q	VUS		2	0.351 Tolerated	0.011 B	15.29 Deleterious
PALB2	c.2474G > C	NM_024675.4	Likely benign	missense_variant	p.R825T	VUS		2	0.043 Tolerated	0.026 B	6.958 Tolerated
PRF1	c.65del	NM_005041.5	Likely Pathogenic	inframe_deletion	p.P22Rfs*29	Likely pathogenic		2	-	-	23.4 Deleterious

D: probably damaging; P: possibly damaging; B: benign; VUS: variants of Uncertain significance

Consistent with the results of the analysis, three functional predictive tools (SIFT, PolyPhen2, CADD) showed genes with clear pathogenicity (*TP53*, *PTEN*, *FCHO1*, *PKM*, *LRP12*). Variants of *TP53* and *PTEN* were confirmed by Sanger sequencing or IHC (supplementary Fig). Variants of unknown significance (VUS) in three genes (*FCHO1*, *PKM*, *LRP12*) identified that have been predicted to be pathogenic.

## Discussion

Malignant PTs of the breast are rare with unclear genetic pathogenesis and progression mechanisms. Malignant PTs in pregnancy and lactation are even rarer, and there are currently no genome sequencing studies on the pathogenesis and progression of such tumors. The present study describes the results of somatic and germline genetic alteration analyses in two patients with malignant PTs diagnosed during pregnancy and lactation.

The average age of onset of PT during pregnancy is 30 years, significantly younger than the typical age of malignant PTs[18]. Gestational PTs may be larger, faster growing, and bilateral than no gestational PT, possibly due to hormonal dependence during pregnancy and lactation[10, 19]. This rare tumor usually has a very aggressive course, and may recur as extensive local or distant disease[10, 19, 20]. There is no specific treatment guideline for such a special tumor, and the literature demonstrates that surgery alone might be insufficient[18, 21]. Other therapy regimens, including chemotherapy, endocrine therapy, and radiotherapy, are not recognized as standard treatments, and their efficacy is controversial[18, 21]. In our study, Patient 1 progressed with adjuvant radiotherapy after surgery, while Patient 2 received no adjuvant therapy after surgery and currently shows no signs of disease progression. Therefore, revealing the tumorigenesis and progression of tumors from a genetic perspective will provide clues for predicting biological behavior and provide a basis for individualized treatment.

Our study applied NGS technologies to assess the genomic alterations and actionable in patients with malignant PTs during pregnancy and lactation. *MED12* is the most commonly mutated gene in breast PTs, and is less frequent in higher pathological grades, suggesting its role in the initiation and early progression of these tumors[22–24]. In addition to *MED12*, *TERT*-promoter, *RARA* and *TP53* variants are frequent in malignant PTs, and one study showed that *MED12* mutations were associated with improved disease-free survival rates and a reduced likelihood of recurrence, and *TP53* mutations were usually associated with tumor grade progression[23, 25, 26].

Compared with the genome of nongestational malignant PTs, our genome sequencing results revealed that the analyzed tumors shared some common alterations with nongestational malignant PTs, including *ATM*, *TP53*, *RB1*, and *PTEN*. Additionally, some genes were detected for the first time in malignant PTs during pregnancy and lactation, but their significance in tumor formation and progression is unknown. Except for the *TP53* mutation, which was present in both patients, the gene aberrations of Patients 1 and 2 were significantly different. It has been suggested that *TP53* mutation may independently promote the pathogenesis of malignant PTs or promote tumor progression based on *MED12* mutation [23, 26–34]. *TP53* is a classic tumor suppressor gene involved in many malignant tumors, often accompanied by advanced tumor grade and poor prognosis, with high proliferative and invasive ability and genomic instability[35]. *TP53* mutation may cause resistance to platinum, fluorouracil and other chemotherapeutic drugs[36, 37], and *TP53* mutations may reduce radiotherapy sensitivity by promoting cell proliferation and metastasis[38, 39]. This may be one of the reasons why Patient 1 progressed after 25 radiation treatments. Many studies have revealed that restoring the wild-type gene state of *TP53* may be a therapeutic target for tumors[38, 39]. Clinical trials have shown that AZD1775 was effective against *TP53* mutated tumor cells as a single agent in combination with chemotherapy agents or in combination with olaparib[38].

In Patient 2, two tumor suppressor gene variants in *PTEN* and *TP53* were predicted to be pathogenic. *PTEN*, which has been previously reported in malignant PTs, is a tumor suppressor mutated in many cancers at high frequency[22, 23, 27, 28]. The protein encoded by this gene negatively regulates intracellular levels of phosphatidylinositol-3,4,5-trisphosphate in cells and functions as a tumor suppressor by negatively regulating the AKT/PKB signaling pathway[40]. Clinical trial results revealed that PI3K inhibitors such as GSK2636771 and AZD8186 showed antitumor activity against *PTEN*-deficient cancers.

Other altered genes identified in our study, including *TGFB1*, *ARHGEF17*, *FCHO1*, *GON4L*, *KRT27*, *TGFB1*, *TTN*, *CFAP57*, *EMILIN2*, *F2R*, *IGSF9B*, *LILRB2*, *LRP12*, *OR5AN1*, *PCDHA13*, *PKM*, *RELN*, *SATB1*, *SF3B3*, *SYT16*, *ZSCAN1*, and *ZXDB*, were not classified as oncogenes or tumor suppressor genes. Among them, *FCHO1*, *PKM*, and *LRP12* were predicted to be probably disease-causing, and only *FCHO1* was previously reported to be a variant in benign PTs [27]. *FCHO1*, implicated in primary immunodeficiency disease, is a member of the Fer/CIP4 homology-Bin/amphiphysin/Rvs (F-BAR) protein family, which contains an F-BAR domain[41]. In addition to its role in benign PTs, *FCHO1*, which regulates cell division, participates in tumorigenesis in leukemia and solid tumors such as lung cancer [42]. This suggests that *FCHO1* might play an important role in the carcinogenesis and progression of PTs. *LRP12* and *PKM*, which are both involved in metabolic pathways, have been reported to participate in carcinogenesis in solid malignant tumors [43, 44]. *LRP12* encoded protein is associated with Oculopharyngodistal Myopathy 1 and Neuronal Intranuclear Inclusion Disease[45, 46]. Among its related pathways are signaling by GPCR and Metabolism of fat-soluble vitamins[45, 46]. *PKM* encoded proteins involved in glycolysis participate in many malignant tumors, especially in *TP53* mutation hepatocytic cell carcinoma[47–49]. Additionally, Pyruvate Kinase Deficiency Of Red Cells is a disease usually associated with *PKM* [50]. All three genes may be associated with tumorigenesis and progression.

Differences at the genomic level between the two patients suggest that there may be different individualized treatment options available. The results of our study and those reported in the literature confirm that a*TP53* mutations are highly frequent in malignant PTs, with a mutation frequency of up to 50%, while the mutation frequency of *PTEN* is lower than that of *TP53*, at approximately 10%[22, 23, 27]. Therefore, these two genes may be potential therapeutic targets for patients similar to ours. In addition, TMB has been extensively investigated as an immune checkpoint inhibitor (ICI) predictive biomarker in different randomized trials[51]. In pancancer studies, a high TMB in up to 20 tumor types correlated with ICI response[51, 52]. In the most recent analysis, ICls had a similar relative benefit compared with chemotherapy in all populations, regardless of whether the TMB was high or low in lung cancer[52]. Numerous early studies have demonstrated significant overlap in the range of TMB between responders and nonresponders [52]. In malignant PTs, the effect of chemotherapy and radiotherapy is controversial[9]. According to the above analysis, even though our study patients had TMBs of 0.3 and 0.5, immunotherapy may be a new treatment option; however, the above assertions require verification in clinical trials.

Germline alterations in many protein-coding genes are pathogenic factors related to cancer predisposition[53]. Reports of germline variants of malignant PTs are very rare, and common germline variation genes associated with breast tumors were not detected in this study, including some germline variation genes related to malignant PTs, such as *TP53*, *PTEN*, *RB1* and *BRCA1*, and *BRCA2*[54]. *PRF1* (Perforin 1) encoded protein plays a key role in secretory granule-dependent cell death, and defenses against virus-infected or neoplastic cells[55, 56]. *PRF1* mutation escape from immune surveillance is a major putative mechanism of tumorigenesis[57]. *PRF1* germline mutations have been associated with an autosomal recessive immune deficiency, familial hemophagocytic lymph histiocytosis of type 2 and childhood anaplastic large cell lymphoma (ALCL) [57]. Our study is the first to identify *PRF1* as a possible germline mutation gene associated with malignant PTs during pregnancy and lactation. More evidence is required to verify that this gene variation is related to tumorigenesis. Identification of a genetic predisposition to developing PT would (1) direct enhanced screening for at-risk and affected women, (2) facilitate conversations regarding risk-reducing strategies for known cancer syndromes (e.g., Li-Fraumeni), and (3) potentially have significant implications for direct relatives, with an opportunity for cascade testing.

## Conclusion

The present study is the first to report the genomic characteristics of pregnancy-related malignant PTs. Analysis by NGS provided new insights into the molecular pathogenesis of such patients, and identified novel alterations involved in the pathogenesis and progression of pregnancy-related malignant PTs. This study identified two potential drug targets (*TP53*, and *PTEN*) and a potential germline variant (*PRF1*).

## Electronic supplementary material

Below is the link to the electronic supplementary material.

Supplementary Material 1

Supplementary Material 2

Supplementary Material 3

## Data Availability

All data generated or analyzed during this study are included in this published article.
