# Correction to: Genomic characteristics of two breast malignant phyllodes tumors during pregnancy and lactation identified through whole-exome sequencing

**DOI:** 10.1186/s13023-022-02583-4

**Published:** 2022-12-05

**Authors:** Ting Lei, Mengjia Shen, Xu Deng, Yongqiang Shi, Yan Peng, Hui Wang, Tongbing Chen

**Affiliations:** 1 Department of Pathology, The Third Affiliated Hospital of Soochow University, Changzhou, 213003 Jiangsu People’s Republic of China; 2 Department of Pathology, West China Hospital, Sichuan University, No. 37, Guo Xue Xiang, Chengdu, 610041 Sichuan People’s Republic of China

**Correction to: Orphanet Journal of Rare Diseases (2022) 17:382** 10.1186/s13023-022-02537-w

Following publication of the original article [1], we have been notified that one of the authors’ names was mentioned incorrectly. The correct name should be as per below:

First name—Ting, last name—Lei.
