# Supplementary figures and images for: Genomic characteristics of two breast malignant phyllodes tumors during pregnancy and lactation identified through whole-exome sequencing
Source: Orphanet J Rare Dis. 2022 Oct 21;17:382. doi: 10.1186/s13023-022-02537-w (PMC9587670; doi:10.1186/s13023-022-02537-w)

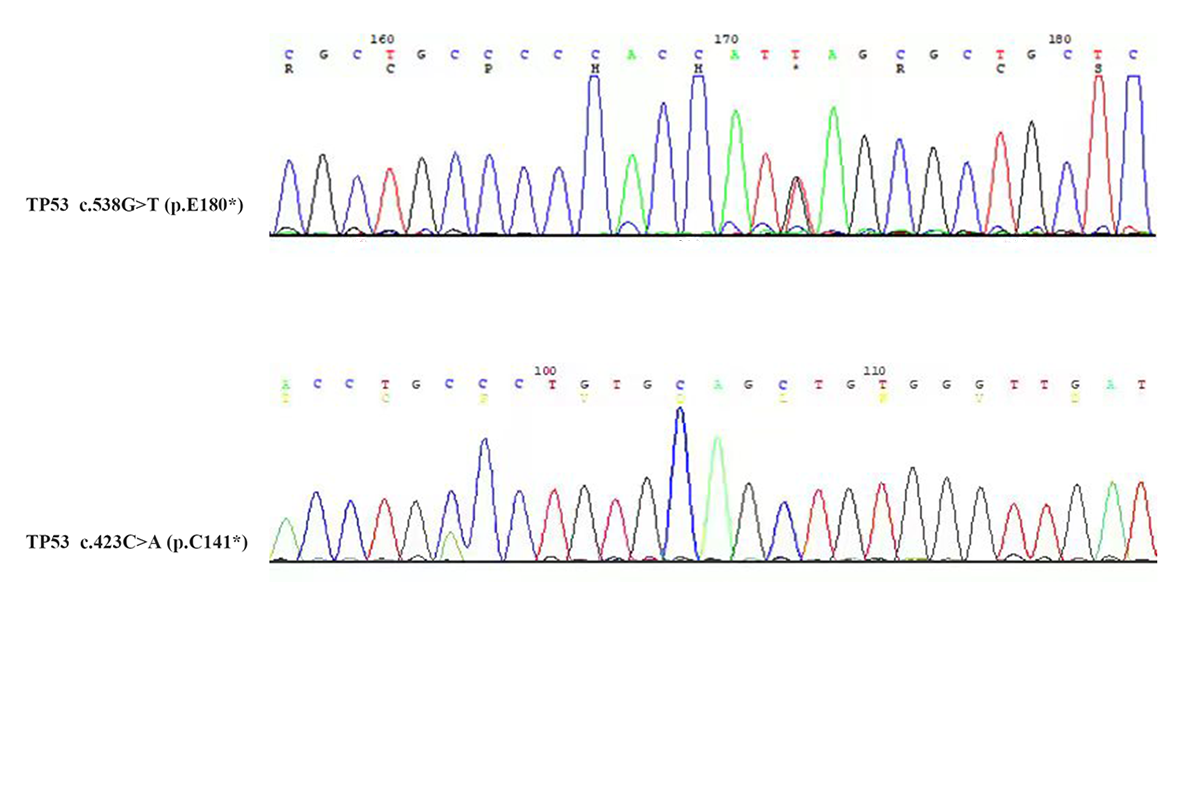

Supplement: Supplementary file 1 — Supplementary Material 1 [file 13023_2022_2537_MOESM1_ESM.tif]

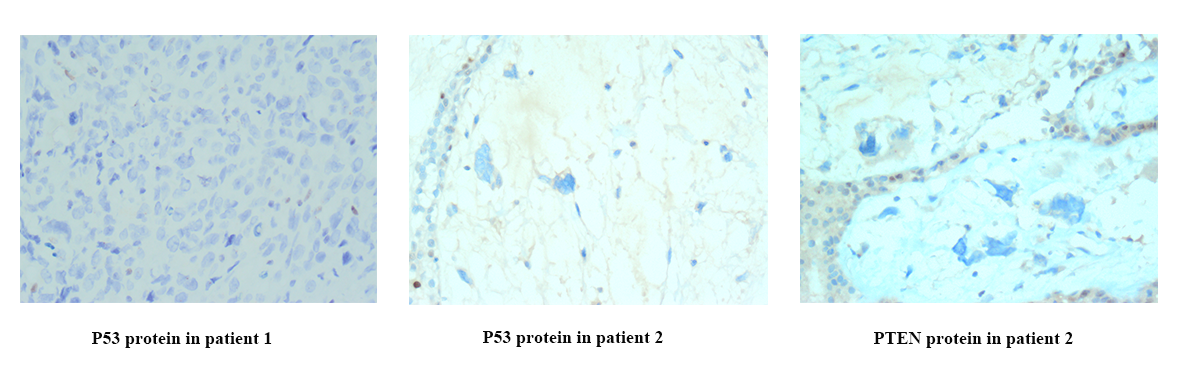

Supplement: Supplementary file 2 — Supplementary Material 2 [file 13023_2022_2537_MOESM2_ESM.tif]
