# Supplementary material for: Genomic characteristics of two breast malignant phyllodes tumors during pregnancy and lactation identified through whole-exome sequencing
Source: Orphanet J Rare Dis. 2022 Oct 21;17:382. doi: 10.1186/s13023-022-02537-w (PMC9587670; doi:10.1186/s13023-022-02537-w)
Supplement: Supplementary file 3 — Supplementary Material 3 [file 13023_2022_2537_MOESM3_ESM.doc]

**Supplementary Information**

| **Additional file: Fig. 1 Sanger sequencing results of TP53 in two patients: Patient 1 (TP53:** **c.423C>A(p.C141*)), Patient 2 (TP53: c.538G>T(p.E180*)). Fig. 2 Immunohistochemistry staining of P53 protein in Patient 1 and Patient 2: P53 protein were absent in neoplastic cells in both samples; immunohistochemistry staining revealed the absence of PTEN protein in the neoplastic cells of Patient 2.** |
| --- |
